# Wild Grape-Associated Yeasts as Promising Biocontrol Agents against *Vitis vinifera* Fungal Pathogens

**DOI:** 10.3389/fmicb.2017.02025

**Published:** 2017-11-03

**Authors:** Gustavo Cordero-Bueso, Nicola Mangieri, David Maghradze, Roberto Foschino, Federica Valdetara, Jesús M. Cantoral, Ileana Vigentini

**Affiliations:** ^1^Department of Biomedicine, Biotechnology and Public Health, University of Cádiz, Cádiz, Spain; ^2^Department of Food, Environmental and Nutritional Sciences, University of Milan, Milan, Italy; ^3^Department of Viticulture and Enology, Institute of Horticulture, Viticulture and Oenology, Agricultural University of Georgia, Tbilisi, Georgia

**Keywords:** yeasts, molds, *V. vinifera* ssp sylvestris, biocontrol, fungal diseases

## Abstract

The increasing level of hazardous residues in the environment and food chains has led the European Union to restrict the use of chemical fungicides. Thus, exploiting new natural antagonistic microorganisms against fungal diseases could serve the agricultural production to reduce pre- and post-harvest losses, to boost safer practices for workers and to protect the consumers' health. The main aim of this work was to evaluate the antagonistic potential of epiphytic yeasts against *Botrytis cinerea, Aspergillus carbonarius*, and *Penicillium expansum* pathogen species. In particular, yeast isolation was carried out from grape berries of *Vitis vinifera* ssp sylvestris populations, of the Eurasian area, and *V. vinifera* ssp *vinifera* cultivars from three different farming systems (organic, biodynamic, and conventional). Strains able to inhibit or slow the growth of pathogens were selected by *in vitro* and *in vivo* experiments. The most effective antagonist yeast strains were subsequently assayed for their capability to colonize the grape berries. Finally, possible modes of action, such as nutrients and space competition, iron depletion, cell wall degrading enzymes, diffusible and volatile antimicrobial compounds, and biofilm formation, were investigated as well. Two hundred and thirty-one yeast strains belonging to 26 different species were isolated; 20 of them, ascribed to eight species, showed antagonistic action against all molds. Yeasts isolated from *V. vinifera* ssp sylvestris were more effective (up to 50%) against *B. cinerea* rather than those isolated from *V. vinifera* ssp vinifera. Six strains, all isolated from wild vines, belonging to four species (*Meyerozyma guilliermondii, Hanseniaspora uvarum, Hanseniaspora clermontiae*, and *Pichia kluyveri*) revealed one or more phenotypical characteristics associated to the analyzed modes of antagonistic action.

## Introduction

Plants provide over 80% of the human diet. Just three cereal crops (i.e., rice, maize, and wheat) and two fruit crops (grape-berries and citrus fruits) provide 70% of energy intake and cope the production of 80% of the fermented beverages in the world (FAO, 2011). Since the 1900s, around 75% of crop diversity has been lost from farmers' fields. Regarding harvest products, many losses (up to 25% of total production in industrialized countries and more than 50% in developing countries) are attributed to decay fungi, such as the *Botrytis, Penicillium, Aspergillus*, or *Cholletotrichum* genera, which are also the source of mycotoxins, harmful compounds to humans (FAO, 2011). The control of fungal diseases and mycotoxins in food and feed chains is principally based on the use of synthetic fungicides. In 2015, Spain, France, Italy, and Germany together made up 70.5% of the European Union-28's pesticide sales. Fungicides are also increasing the level of hazardous residues in the environment, they are becoming less effective due to both the increasing of resistant fungal strains, and the use of restrictions carried out by the European authorities (Directive 2009/128 /EC). Natural diversity and ecosystems provide agricultural production in many different ways (Power, 2010), but not all are well-known. Although animal and plants have received considerable attention as a resource for natural-product discovery, the microbiological component of this natural richness remains relatively unexplored.

Yeasts are unicellular fungi that have been isolated from different ecosystems and sources both natural and in connection with human activities. They can be found on/in fruits, including *Vitis vinifera* ssp vinifera cultivars and *V. vinifera* ssp. sylvestris, plants, insects, animal intestinal tracts, soils, and marine environments (Kurtzman et al., 2011). In the past 35 years, there have been extensive research activities to explore and develop the potential of yeasts as antagonists to biologically control harvest pathogens and as an alternative to chemical pesticides (Liu et al., 2013). Representing an eco-friendly alternative to synthetic pesticides, the use of antagonist yeasts as biocontrol agents has generated a great enthusiasm (Wisnieswski et al., 2007; Droby et al., 2009; Sipiczki, 2016; Spadaro and Droby, 2016). However, yeasts often show a lower and non-comparable effectiveness against pathogenic fungi (*Botrytis cinerea, Aspergillus carbonarius, and Penicillium expansum*) in comparison to chemical fungicides (Liu et al., 2013), thus reducing their practical applications and leaving the problem of plant fungal disease still unsolved. Considerable progress has been made in increasing knowledge and commitment to elucidate some modes of action of few yeast strains against pathogenic fungi (Sipiczki, 2006; Sharma et al., 2009; Jamalizadeh et al., 2011; Spadaro and Droby, 2016). The described mechanisms are; nutrient or space competition (Suzzi et al., 1995), iron depletion (Sipiczki, 2006; Parafati et al., 2015), extracellular lytic enzymes production (Bar-Shimon et al., 2004), volatile organic compounds (Fredlund et al., 2004), reactive oxygen species (ROS) tolerance (Jamalizadeh et al., 2011; Liu et al., 2011), biofilm formation (Giobbe et al., 2007; Wisnieswski et al., 2007), or inducing host-plant resistance throughout the accumulation of phytoalexins (Arras, 1996; Jeandet et al., 2002) and the synthesis of pathogenesis-related proteins (Chan and Tian, 2006). Inhibition capabilities on mycelial growth or conidia germination in molds have been reported by some yeast strains of species living in vineyards, overwintering grapes, and cellar ecosystems (Elmer and Reglinski, 2006; Nally et al., 2012; Sipizcki, 2016). Nevertheless, all the scientific strategies focused on looking at different components of such interactions separately or taking into consideration binary or ternary trophic levels of the host-pathogen-antagonist interplay (Droby et al., 2009; Spadaro and Droby, 2016). In general, interactions are not between two single microorganisms and the host; they also involve the native microbiota of the host and the environmental factors (i.e., the variation of the climatic conditions and other abiotic factors such as the soil, plant emplacement, or nutrient availability for the plant). In the case of the vineyards, efforts to understand the influence of different agronomic parameters on yeast populations associated to grape-berries have been published (Cordero-Bueso et al., 2011a,b, 2014) but there is still a lack of bibliography. Moreover, there are unexplored ecosystems such as wild vines like the protected species *V. vinifera* ssp sylvestris (Gmelin) Hegi which could represent a great reservoir of novel and promising yeast species to be used in the food industry, as well as a substitutive of agrochemicals.

The main aim of this work was to evaluate the antagonistic potential of yeasts isolated from grape berries collected from *V. vinifera* ssp sylvestris populations in the Mediterranean and Black Sea basins and from *V. vinifera* ssp vinifera cultivars managed under three different farming systems: organic, biodynamic, and conventional. The mode of action and the grape-berry population associate to grape-berries were investigated as well.

## Materials and methods

### Yeast strain identification

**Figure 1:**
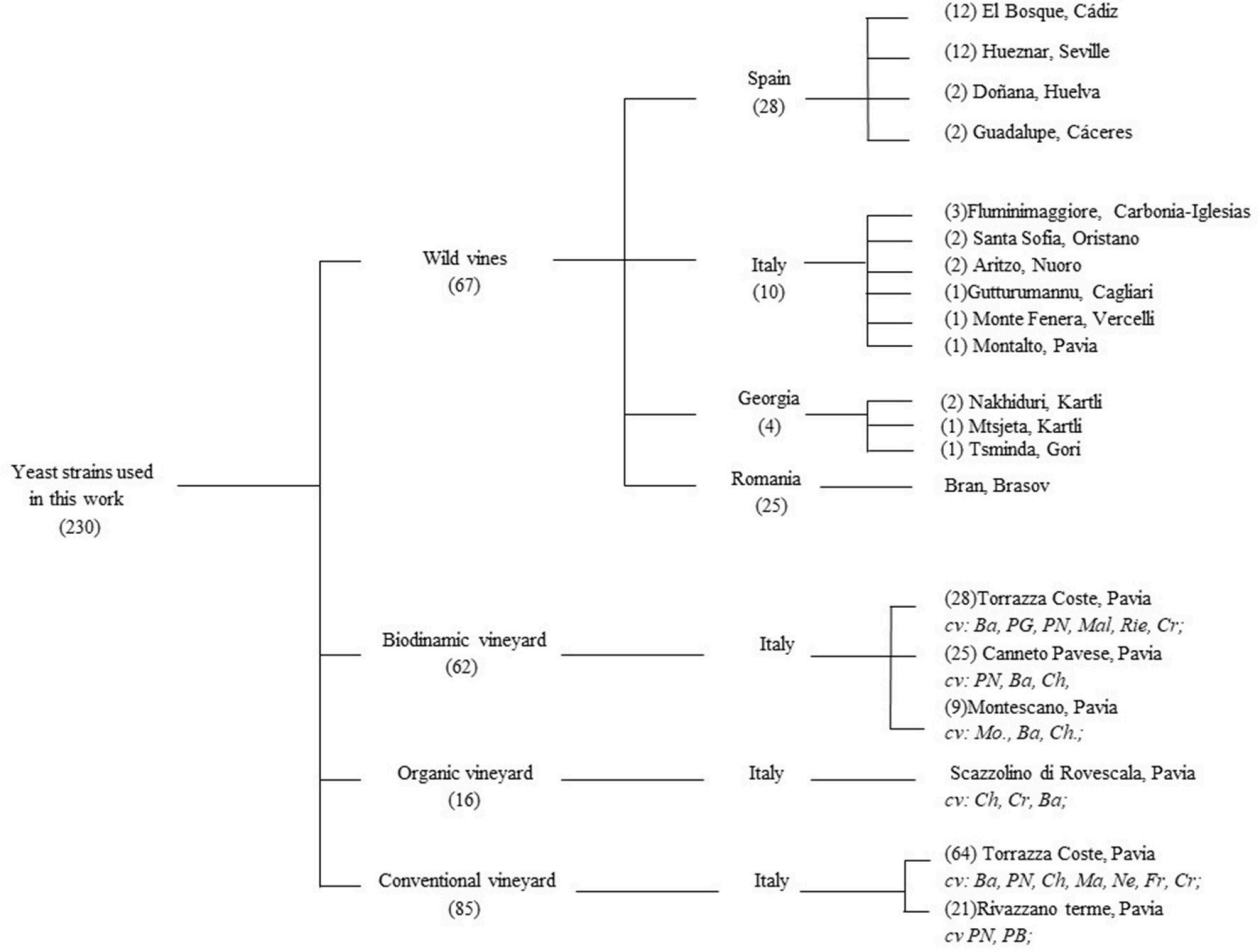
Origin and source of the yeast strains assayed in this work.

Yeast strains were isolated between 2013 and 2016 from grape berries collected in Georgia, Italy, Romania, and Spain from *V. vinifera* ssp. sylvestris populations as stated in Cordero-Bueso et al. (2017) and in Italy from *V. vinifera* ssp. vinifera cv. Pinot Noir cultivated in three different farming systems: organic, biodynamic, and conventional in 2014 (Figure 1). Grape samples were treated following the protocol of Vigentini et al. (2016). All yeasts used in this work were stored in YPD medium (20 g/L peptone, 10 g/L yeast extract, 20 g/L glucose) added with 20% (v/v) glycerol at −80°C. Fresh yeast cultures were obtained by inoculation 1% (v/v) glycerol stocks in YPD broth at 25°C for 3 days in aerobic conditions. Isolates were also plated onto Wallerstein Laboratory Nutrient Agar (WL) to evaluate colony diversity as suggested by Pallmann et al. (2001). DNA extraction from the yeast isolates was performed according to Querol et al. (1992). The patterns belonging to the different species were obtained by Restriction Fragment Length Polymorphism (RFLP) analysis of the amplified ITS1-5.8S-ITS2 region; the primers used for DNA amplification were ITSY1 (5′-TCCGTAGGTGAACCTGCGG-3′) e ITSY4 (5′-TCCTCCGCTTATTGATATGC-3′) as described by White et al. (1990). PCR products were digested by *Cfo*I, *Dde*I, *Hae*III, and *Hinf* I restriction enzymes (Thermo Fisher Scientific, Massachusetts, U.S.A.). *Meyerozyma guilliermondii* (anamorph *Candida guilliermondii*) and *Meyerozyma caribbica* (anamorph *Candida fermentati*) are closely related species. Thus, to avoid misidentification these species of yeasts were also subjected to RFLP analysis using the enzyme *Taq*I as stated by Romi et al. (2014). Amplification products and their fragments were separated on 1.4% (w/v) and 2.5% agarose gel, respectively, added with 0.05 μg/L of ethidium bromide in TAE buffer (Tris-acetate 40 mM, EDTA 1 mM, pH 8) at 100 V for 90 min. The agarose gels were visualized using UV and photographed (1000 System, Bio-Rad Laboratories, California, U.S.A.). At least two representative members from each ITS-RFLP genotype group were randomly selected for sequencing LSU sRNA gene D1/D2 domain. Certain database sequences of several species such as *Aureobasidium pullulans* and *Rhodotorula nothogafi*, have identical D1/D1 sequences with other species. Thus, when necessary, we included the ITS1-5.8S-ITS2 region sequences. Amplification of D1/D2 region was carried out using primers NL1 (5′-GCATATCAATAAGCGGAGGAAAAG-3′) and NL4 (5′-GGTCCGTGTTTCAAGACGG-3), as previously described Kurtzman and Robnett (1998). Purification and sequencing of PCR products were performed by Macrogen Inc. facilities (Seoul, South Korea) using an ABI3730 XL automatic DNA Analyzer. The obtained sequences were aligned using ClustalX algorithm. The Basic Local Alignment Search Tool (BLAST) (http://www.ebi.ac.uk/blastall/nucleotide.html) was used to compare the sequences obtained with databases from the European Molecular Biology Laboratory (EMBL). As proposed Sipiczki (2016), the sequences of the strain types were also determined by pairwise Blast alignment using the bl2seq algorithm available at the website of the NCBI (http://www.cbs.knaw.nl). We considered an identification as “correct” when the gene sequence showed an identity ≥ 98% and a good query cover with the exception of the species *Vishniacozyma carnescens* and *V. victoriae* which D1/D2 sequences of their type strains differ only by 1.8%. Moreover, yeast strains were tested for the fermentation or assimilation of the different compounds as sole carbon, nitrogen, and others sources, with the exception of the hexadecane, vitamin-free, 5-keto-D-glucanase, saccharate, cadaverine, and CoQ component, as stated in Kurtzman et al. (2011) but using a 96-well microtiter plate technology.

### Mold strains and growth conditions

The mold strains used in this work were *P. expansum* UCAF0034 (Colección de la Universidad de Cádiz, Spain), *B. cinerea* BO5.10 (*Colección Española de Cultivos Tipo*, Burjassot, Valencia, Spain), and *A. carbonarius* UCAF0012 (Colección de la Universidad de Cádiz, Spain). Molds were selected based on their virulence by artificial inoculation on wounded grapes (data not shown).Mold cultures were plated on a Potato Dextrose Agar medium (Conda Laboratories, Torrejón de Ardoz, Madrid, Spain). Plates were incubated at 25°C under constant white light for at least 10 days. After incubation, spores were collected in a solution of 0.1% (v/v), Tween 20 (SIGMA). The concentration of the conidial suspension was adjusted to give 6 × 10^6^ spores/mL according to Comménil et al. (1999). Mold strains were stored as conidial suspensions added with 20% (v/v) glycerol at −80°C.

### *In vitro* assays for antagonistic activity

#### Dual screening of antagonistic activity on agar media

The antagonistic activity of the 241 yeast isolates against *A. carbonarius, B. cinerea*, and *P. expansum* molds was investigated by *in vitro* assay. In the *first* screening, 5 μL of a fresh conidial suspension of the molds, one for each plate, were inoculated in the center of the PDA plate. Then, 5 μL of six fresh yeast cultures were positioned at 2.5 cm from the center of each Petri dish. The plates were incubated at 25°C for 10 days under constant white light and 80% relative humidity. A clear zone around the yeast colonies was interpreted as total inhibition of the growth of the mold. The strains showing an inhibitory activity were chosen for the second step of selection. In this case the PDA plates were prepared as follows: 10 mL of PDA were first included in each plate; afterwards, 5 mL of soft PDA (7 g/L agar) containing a final concentration 10^6^ CFU/mL of yeast cells, one for each strain, were inoculated in the plates. Subsequently, when the plates were solidified, 5 μL of fresh conidial suspensions of the tested molds were inoculated upon them. The plates were incubated at the same conditions of first screening. After incubation, the radial growth was measured and the inhibition percentage was calculated as follows: inhibition (%) = (DC – DA)/DC x 100, where DC is the diameter of the growth area without the antagonistic yeast (control), DA is the diameter of growth area with the antagonistic yeast (Ruiz-Moyano et al., 2016). The experiments were repeated three times to confirm reproducibility of the results.

#### Evaluation of the minimum inhibiting concentration

An estimation of the starting concentration of yeast cells capable to inhibit the mold growth was carried out by the following test. Fresh cultures of the yeasts that overcome the second step of selection were grown in YPD broth at 25°C for 3 days. PDA plates were prepared for each strain containing a different cell concentration, from 10^3^ to 10^6^ CFU/mL. When the plates solidified, 10 μL of conidial suspensions (3 × 10^5^ spores/mL) of *B. cinerea, A. carbonarius*, and *P. expansum* were spotted on the center of the Petri dish. The plates were incubated at 25°C for a week under constant light. The results were considered positive when the yeast was able to inhibit the total mold growth within the time of incubation. Control tests without inoculated yeast cells were carried out. The experiments were repeated three times to confirm reproducibility of the results.

#### Killer character assay

The killer character assay was performed according to Stumm et al. (1977). Plates containing YPD-agar and 0.003% (w/v) of methylene blue that was buffered to pH 4.5 with 0.1 mol/L of citrate-phosphate buffer were used. Yeast strains were cultured in liquid YPD until their exponential growth phase. Then, yeast strains were diluted in YPD and spread onto the plates at a concentration of 10^5^ cells per plate and incubated at 25°C for 48–96 h. Killer activity was scored positive when the killer strain was surrounded by a region of bluish-stained cells, or by a clear zone of growth inhibition bounded by stained cells.

#### Test for lytic enzymes activity

In order to investigate the reason of the observed inhibitory effect, the previous selected strains were examined taking in consideration the production of cell wall lytic enzymes. Yeast fresh cultures were adjusted at a final concentration of 1 × 10^6^ CFU/mL. To evaluate the proteolytic activity, 20 μL of the yeast suspension were spotted onto Skim Milk agar (Merck, Darmstadt, Germany); the formation of a clear halo around the colony after incubation at 25°C for 5 days indicated the enzymatic activity. Glucanase and chitinase activities were determined by replica plating technique. In this case, 20 μL of the yeast suspension were spotted onto YPD plates containing 0.2% β-glucan (Sigma, Town, Nation) and YPD plates containing 0.2% chitin (Sigma). Petri dishes were incubated at 30°C for 5 days. Colonies were rinsed off the plates with distilled water before staining the plates with 0.03% (w/v) Congo Red. A clear zone around the colony meant the presence of glucanase activity. Yeasts were screened for polygalacturonase production with the method described by Strauss et al. (2001) as well; they were spotted onto polygalacturonate Agar Medium containing 12.5 g/L polygalacturonic acid (Sigma), 6.8 g/L potassium phosphate (pH 3.5), 6.7 g/L yeast nitrogen base without ammonium sulfate (YNB, Difco), 10 g/L glucose, and 20 g/L agar. Plates were incubated at 30°C for 5 days. Colonies were rinsed off the plates with deionized water before staining the plates with 0.1% (w/v) Ruthenium Red. Colonies showing a purple halo were considered positive. β-glucosidase activity was tested by plating the yeast onto a selective medium containing 6.7 g/L yeast nitrogen base (YNB, Difco), 5 g/L arbutin (Sigma), and 20 g/L agar (pH 5.0). Two milliliters of a filter-sterilized 1% (v/v) ammonium ferric citrate solution was added to 100 mL media before pouring onto the plates. Petri dishes were incubated at 30°C for 3 days. Positive colonies were identified by the discoloration of the media to a brown color.

#### Production of volatile organic compounds (VOCs) and hydrogen sulfide release

Selected yeast strains were also evaluated for their production of VOCs and hydrogen sulfide released against the molds *B. cinerea, A. carbonarius*, and *P. expansum*. Four-part Petri dishes containing 3.5 mL of PDA for each sector were used. In one part, 20 μL of 10^6^ CFU/mL of yeast suspension were inoculated. The plates were incubated at 25°C for 3 days. Then, 20 μL of conidial suspension (6 × 10^6^ spores/mL) of each mold were inoculated in the other three sectors of each plate. Plates without the inoculation of yeasts were utilized as control. Finally, the plates were double wrapped with sterile HDPE film (Parafilm, Neenah, U.S.A) to prevent air escape and incubated for 3 days at 25°C under constant white light. Radial growth reduction, in relation to the control test, was calculated after 6 days. All experiments were performed in triplicate. Data were analyzed by one-way ANOVA. The means were separated at the 5% significance level using Tukey's test. The yeast strains slowed or inhibited the mold growth were also tested for the production of acetic acid and hydrogen sulfide. Ten microliters of yeast cell suspensions (10^6^ CFU/mL) were spotted on Biggy Agar (Oxoid, Bakingstoke, U.K.) and in a CaCO_3_ agar medium (5.0 g/L yeast extract; 20 g/L glucose; 10 g/L CaCO_3_; 20 g/L agar). The plates were incubated at 30°C for 3 days. The qualitative amount of H_2_S production on this indicator medium was determined by the color of the colonies, which ranged from white (no release) through brown to near black, depending on the extent of production (high release). In the case of the acetic acid production, a clear zone around the colony meant the presence of acetic acid. A halo greater than 3 mm of radius meant a high acid release, if the halo was between 2 and 3 mm meant low acid release, if the halo was between 1 and 2 mm meant slight acid formation, and if the halo was less than 1 mm meant traces.

#### Biofilm formation

The capability to produce biofilm was evaluated following the protocol of Jin et al. (2003) partially modified. Ten microliters of fresh yeast suspension as previously described were inoculated in 1 mL of Yeast Nitrogen Base (YNB, Difco, Swedesboro, U.S.A.) added with 100 mM glucose and incubated overnight at 28°C. Subsequently, the tubes were centrifuged at 4,000 rpm for 5 min (Rotina 380 R, Hettich Zentrifugen, Tuttlingen, Germany), the cells were washed twice with a 1X phosphate-buffered saline (10X PBS: NaCl 1.37 M, KCl 27 mM, Na_2_HPO_4_ 100 mM, KH_2_PO_4_ 18 mM), pH 7.2) and re-suspended in YNB + glucose (100 mM) medium to obtain 10^7^ CFU/mL. A control test was prepared with the medium without yeast cells added. One hundred microliters of the cell suspension were inoculated in triplicate into 96-well polystyrene plate with flat bottom (Starlab, Hamburg, Germany) at 28°C in a shaker at 75 rpm for 3 h. After the adhesion phase, the wells were washed twice with 150 μL of PBS, and then 100 μL of same medium were added into each well and incubated at 28°C in a shaker at 75 rpm for 72 h. The medium was sucked up daily and, then, 100 μL of fresh YNB were put into each well. After incubation, the wells were washed twice with 150 μL of PBS then 100 μL of crystal violet 0.4% (w/v) were put into each well. After 45 min, the wells were washed again for four times with 150 μL of distillate sterile water and immediately 200 μL of 95% (v/v) ethanol were added. After 45 min, 100 μL of solution were transferred to a new polystyrene 96-well plate and then the solution was measured at 590 nm. The absorbance values were subtracted for the control test values.

#### Effect of iron concentration on the inhibitory activity of the yeast strains

In order to investigate the influence of iron concentration on the inhibitory activity of the selected yeasts the following test was carried out. PDA plates without added iron and plates with 5 and 20 μg/mL of FeCl_3_ were prepared spreading on plates a conidial suspension (3 × 10^5^ spores/mL) of *B. cinerea, A. carbonarius*, and *P. expansum*. Then, 10 μL of yeast suspensions (10^6^ CFU/mL) were dropped on Petri dishes in triplicate. Three plates for each mold without yeast addition were used as control. The plates were incubated at 25°C for 1 week under constant white light. The width of reddish halos developing around the yeast colonies were measured according to Parafati et al. (2015). The results of the role of competition for iron on the antagonistic activity of the yeasts were obtained measuring the width of inhibition zones around the yeast colonies after a week.

#### Effect of other metabolites released by yeast strains on mold growth

In order to examine the effect of other potential metabolites derived from the primary or secondary metabolism of yeasts produced by antagonistic yeasts, the molds were grown in a medium containing the supernatant of a yeast culture. The yeast cultures were grown in 50 mL YPD broth at 25°C for 5–7 days in a shaker at 125 rpm. The cell growth was monitored by spectrophotometer measurements at 600 nm (Jenway 7315, Staffordshire, U.K.). When yeast cultures attained the stationary phase the supernatants were collected by centrifugation at 3,500 rpm for 5 min at 4°C (Rotina 380 R, Hettich Zentrifugen, Tuttlingen, Germany) and filtered by a 0.45 μm sterile membrane (Minisart, Goetting, Germany). Five, 0.5, and 0.05 mL of supernatants were mixed with warm (<45°C) and concentrated 5X PDA medium by adjusting the volume with sterile distilled water and poured in Petri dishes. When the plates solidified, 10 μL of conidial suspensions (3 × 10^5^ spores/mL) of *B. cinerea, A. carbonarius*, and *P. expansum* were inoculated. The plates were incubated at 25°C for a week under constant light. The test was considered positive if the tested molds did not grow or if a severe growth inhibition was observed with respect to the control.

### *In vivo* assays for inhibitory activity

#### Efficacy of yeast strains in controlling grapes infected by molds

The yeast strains showing an evident inhibitory activity by *in vitro* assays were selected for the *in vivo* test. Fresh yeast cultures were collected by centrifugation at 3,000 rpm (Rotina 380 R, Hettich Zentrifugen, Tuttlingen, Germany) for 5 min at 4°C and washed twice with sterile distilled water. The yeast suspensions were adjusted at 10^6^ CFU/mL. Healthy berries of table grapes (cultivar Superior Seedless, Egypt) were used for the test. Grape berries surface was disinfected by dipping them in a solution 1% (v/v) sodium hypochlorite for 5 min and rinsed three times with sterile distilled water. Afterwards, three berries for treatment were cut with a sterile scalpel (one wound of 5 mm for each berry) and submerged in the yeast cells suspensions for 5 min. The berries were put into sterile 50 mL Falcon tubes (Sigma-Aldrich, Darmstadt, Germany) and incubated for 24 h at 25°C. Then, the wounds were inoculated with 20 μL of conidial suspension (6 × 10^6^ spores/mL) of *B. cinerea, A. carbonarius*, and *P. expansum* (three berries for each mold and for each yeast) and incubated at 25°C under constant light for a week. Three berries for each mold without yeast cells were used as control. The disease severity was evaluated by a visual score “1-to-4” (1: no visible symptoms; 2: soft rot; 3: formation of mycelium; 4: sporulation of mold) according to Parafati et al. (2015).

#### Inhibitory effect of yeasts vs. a chemical pesticide by *in vivo* tests

The inhibiting activity of strains, that showed the best results in the previous tests, were compared to the commercial pesticide Switch®, Syngenta (37.5% *Cyprodinil* and 25% *Fluodioxinil*). The fresh yeast cultures were prepared as above described. The pesticide was used at the suggested concentration of 1 g/L, according to the manufacturer's instruction, and it was dissolved in 25 mL of distilled sterile water. Healthy berries of table grape (cultivar Sugarone, Chile) for each yeast strain, pesticide, and control, repeated for the three tested molds, were used in this trial. The berries were treated and disinfected as above described. Afterwards, the berries were submerged in the solutions containing the yeast cells and in the solution containing the chemical pesticide for 5 min. Three berries for each mold without yeast cells and pesticide were used as control. The berries were included in six-well plate (Starlab, Hamburg, Germany) at 25°C for 24 h. Then, 10 μL of conidial suspension (6 × 10^6^ spores/mL) of *B. cinerea, A. carbonarius*, and *P. expansum* were inoculated on the berries, in the correspondingwound points. The plates were incubated at 25°C for a week under constant light. The results were evaluated by a visual score previously stated.

## Results

### Identification of yeasts

Two hundred and thirty-one yeast strains were isolated from grape berries samples of different vines: 85, 62, and 16 from a conventional, a biodynamic, and an organic vineyard, respectively. Sixty-seven yeasts were collected from *V. vinifera* ssp. sylvestris. The sampling plan and the distribution of the isolates are reported in Supplementary Material 1. Sixteen different morphologies were observed on WL-agar plates (data not shown). Three distinct colony subtypes were also identified within the pink-halo producers. Molecular identification by using amplification and restriction analysis of ITS1-5.8S-ITS2 region revealed 26 different patterns. The D1/D2 region of the 26S rDNA gene of at least two yeast strains, for each potential species was sequenced to identify the species. Table 1 shows the number of strains ascribed to each different species. The accession number of the sequences deposited at GenBank and the most similar CBS strain numbers are shown in Tables 1, **3**. *Aureobasidium pullullans* can easily be confused with *Aureobasidium subglaciale, Kabatiella microsticta*, or *Columnospaeria fagi* because many database sequences of these species have identical D1/D2 sequences (Brysch-Herzberg and Siedel, 2015; Sipiczki, 2016). Moreover, *R. nothofagi* is difficult to distinguish from *C. pallidicorallinum* because certain database of sequences of these species have identical D1/D2 sequences (Sampaio, 2011; Sipiczki, 2016). Therefore, we analyzed the ITS region of *A. pullulans* and *R. nothofagi* as well (Table 1). Since mating partners of the type strains of these species exhibited the most similar ITS sequences and the most similar D1/D2 sequences it's justified to assign the yeast strains of this study to *A. pullulans* and *R. nothofagi*. Furthermore, our strain of *R. nothofagi* did not grow on maltose, trehalose, and inulin, which are usually assimilates by *C. pallidicorallinum* (Sipiczki, 2016). The D1/D2 sequence of our strain identified as *V. carnescens* totally fits with the sequences of type strains found in the explored databases.

**Table 1:** Yeast species occurrence and distribution of the isolated and identified from *V. vinifera* ssp sylvestris and from the different vine cultivars of *V. vinifera* ssp vinifera (conventional, biodynamic, and organic), GenBank accession numbers of the deposited sequences and The Centraalbureau voor Schimmelcultures (CBS) and D1/D1 Genbank accession numbers of the most similar types.

**Isolate**			**Most similar type/reference strain**	**Source**				
**Strain code**	**D1/D2 accession no**.	**ITS accession no**.	**Taxonomic name**	**D1/D2 accession number**	**Conventional vineyard**	**Biodynamic Vineyard**	**Organic Vineyard**	***Vitis vinifera* ssp. sylvestris**
FZ02	MF926292	MF783894	*Aureobasidium pullulans* CBS584.75	KT361587.1	46	15	9	1
CABMC2A	MF927682	MF770161	*Candida californica* CBS989	KY816896	–	–	–	1
FZ03a	MF783064	–	*Filobasidium stepposum* CBS10265	KY107724.1	2	–	–	–
HB09c	MF783066	–	*Filobasidium wieringae* CBS1937	KY107733	–	–	–	1
CABMB1A	MF783060	–	*Hanseniaspora clermontiae* CBS8821	EU272040	–	–	–	1
HURM6B	MF926297.1	–	*Hanseniaspora* ssp CBS276	KY107853	–	–	–	4
CAMB9A	MF783054	–	*Hanseniaspora uvarum* CBS9790	KJ794689	17	34	1	28
NUR3AM	MF926296	–	*Hyphopichia pseudoburtoni* CBS2455	KU609072	–	–	–	1
ROMA10^*^	MF783057	–	*Metschnikowia fructicola* CBS8853	AF360542	–	–	–	5
CABM7C^*^	MF783068	–	*Metschnikowia pulcherrima* CBS5833	JN083816	9	8	1	1
CABM9C^*^	MF783069	–	*Metschnikowia* spp CBS5536	KM350710	–	–	–	5
ROMAM1A^*^	MF783062	–	*Metschnikowia viticola* CBS9950	KC859919	–	–	–	2
SEHMA2	MF783056	–	*Meyerozyma caribbica* CBS2829	KX507035	–	–	–	1
SEHIB8	MF783055	–	*Meyerozyma guilliermondii* CBS8105	KY108543	–	–	–	4
HB01a	MF926291	MF783893	*Papiliotrema flavescens* CBS942	AB035042	4	–	1	–
CABM8C	MF926294	MF783895	*Pichia fermentans* CBS5663	EF550234	–	–	–	1
SEMA6B	MF783059	–	*Pichia kluyveri* CBS7274	KY108823	–	–	–	4
SEHM2A	MF927685	MF783892	*Rhodosporidium babjevae* CBS322	AF387771	–	–	–	1
EP02c	MF783058	MF927679	*Rhodotorula glutinis* CBS2889	KY109044	3	4	1	–
HURM4A	MF783067	MF927680	*Rhodotorula mucilaginosa* CBS482	KY109140	–	–	–	1
SEHUM7B	MF783065	MF784281	*Rhodotorula nothofagi* CBS9091	AF444736	–	–	–	1
ARIM1B	MF926295	MF783896	*Rhodotorula paludigena* CBS4477	KY109146.1	–	–	–	1
CABMA3A	MF783053	–	*Saccharomyces cerevisiae* CBS2963	KF214442	–	–	–	1
SEHM1C	MF770267	–	*Scheffersomyces stipitis* CBS7126	KY109584.1	–	–	–	1
PIEM5B	MF783061	–	*Schwanniomyces polymorphus* CBS6456	KY109627	–	–	–	1
HB02b	MF926293	MF783891	*Vishniacozyma carnescens* CBS973	AB035054	4	1	3	–
Total:					85	62	16	67

* *This table shows the most probable yeast strain according to the compared databased belonging to the Metschnikowia clade, but these yeast strains cannot be assigned unequivocally to one of the species in the clade*.

Unfortunately, we encountered the problem that isolates ROMA1A, ROM10, CABM7C, and CABM9C (Table 1) which seem to belong to *Metschnikowia*-like strains, did not show sequence identity of their D1/D2 to any of the type strains despite they were fairly similar to one species of the *Metschnikowia pulcherrima* clade. It happened also with the ITS sequences. In agreement with Lachance (2011), Sipiczki et al. (2013), Brysch-Herzberg and Siedel (2015), Lachance (2016), and Sipiczki (2016), species belonging to the *M. pulcherrima-like* strains cannot be unequivocally assigned to one of the species of this clade after rDNA analysis because some species such as *M. fructicola* or *Metschnikowia andauensis* have a non-homogenized rDNA array. Moreover, these yeast strains cannot be easily separated by phenotypical and physiological tests. Efforts to clarify the taxonomic situation of the *Metschnikowia* clade are required. Although was impossible to assign our strains to one of the currently described species in the *M. pulcherrima* group, we showed in Tables 1, **3**, the most probable species related to this genus according to the results obtained after the analysis performed.

### *In vitro* tests

#### *In vitro* dual assays to show the antagonist yeast-mold interactions

All yeast isolates were subjected to a preliminary *in vitro* assay for the detection of an antagonistic activity against *B. cinerea, P. expansum*, and *A. carbonarius*. Sixty out of the 231 yeast strains showed an effect of slowing down or inhibiting growth of the three tested molds. Thirty-six out of 60 selected antagonistic yeasts were isolated from *V. vinifera* ssp. sylvestris, 9 from the biodynamic vineyard, 1 from the organic vineyard, and 4 from the conventional one (Table 2). The majority of the strains with antagonistic activity were isolated from wildlife vines (53%), followed by those isolated from the biodynamic (14.5%), the organic farming system (6.2%), and the conventional (4.7%) vines (Table 2).

**Table 2:** *In vitro* dual assays of yeast strains against mycelial growth of *B. cinerea, P. expansum*, and *A. carbonarius*.

**Source**	**Isolates from grapes**	**Isolates with inhibitory capacity at preliminary *vitro* assaying**	**% of isolates with inhibitory capacity at preliminary *vitro* assaying**	**Isolates with inhibitory capacity at second *vitro* test**	**% of isolates with inhibitory capacity at second *vitro* test**	**% of isolates with inhibitory capacity**
Wildlife vines	67	42	62.7	18	42.9	26.9
Biodynamic vineyard	62	11	17.7	2	18.2	3.2
Organic vineyard	16	1	6.2	0	0	0
Conventional vineyard	85	6	7.1	0	0	0
Total isolates	230	60	26.1	20	33.3	8.7

*In the first in Vitro assaying, all isolates are present. At second in Vitro test only the positive at first are shown*.

After the preliminary assay, a second *in vitro* test was performed. It consisted of a test on solid medium where Petri-dishes were plated with a yeast cell-top agar suspension and the mold spores were spotted on the center of the plate. The percentage of the mycelium growth was calculated for each yeast strain against each mold (Table S1, Supplementary Material 1). Twenty yeast strains (plus the control) out of 60, which passed the first screening, inhibited the 100% of hyphal growth of the three tested molds in comparison with the control. Among these, 18 strains were isolated from the wild vines and belonged to *H. uvarum* (9), *M. guilliermondii* (2), *P. kluyveri* (2), *S. cerevisiae, H. clermontiae, M. fructicola-*like yeast strain, *M. viticola*, and *C. californica* species, and two strains were isolated from the biodynamic vines and were ascribed to *A. pullulans* and *V. carnescens* species (Table 2). These 20 yeast strains were selected for the successive tests in order to understand the nature of antagonistic activities.

#### Evaluation of the minimum inhibiting concentration (MIC)

MICs were determined in triplicate for all yeast strains selected after dual assays against the different molds. The evaluation of the MIC revealed that the 20 yeasts significantly reduced the progress of hyphal growth of *B. cinerea* and *P. expansum* at a concentration of 10^5^ cells/mL, and 10 (5 *H. uvarum*, 1 *P. kluyveri*, 1 *M. guilliermondii*, 1 *H. clermontiae*, and 1 *S. cerevisiae*) at a concentration of 10^3^ cells/mL both under the mentioned growth conditions (Table 4). However, the occurrence of *A. carbonarius* was completely reduced by only 14 yeast strains at a concentration of 10^6^ cells/mL. Only two yeast strains (1 *H. uvarum* and 1 *S. cerevisiae*) were able to protect grapes or to compete for the nutrients against *A. carbonarius* at a concentration of 10^3^ cells/mL and under the same growth conditions of *B. cinerea* and *P. expansum* (Table 4). The yeasts that were able to protect grapes or to exhaust the medium from all the assayed molds were those isolated from *V. vinifera* ssp. sylvestris.

**Table 3:** Phenotypical assaying for yeast antagonistic activity against molds and their volatile organic compounds (VOCs) referred to mycelial growth reduction of *B. cinerea, P. expansum*, and *A. carbonarius*.

**Species**	**Strain**	**D1/D2 Accession no**.	**VOCs^a^ (%)**	**Protease**	**Pectinase**	**Glucanase**	**Chitinase**	**Glucosidase**	**Killer activity**	**Acetic acid production^b^**	**H_2_S released**	**Iron depletion^c^**	**Biofilm formation^d^**
*A. pullulans*	FZ02a	MF926292	28.0	–	+	+	+	–	–	0.3	+	Positive with *Botrytis*	0.110
*C. californica*	CABMC2A	MF927682	45.0	–	–	–	–	–	–	0	+	Positive with *Botrytis*	0.030
*H. uvarum*	SEHMA6A	MF783054	31.0	–	+	–	–	–	–	0	–	Positive with *Botrytis*	0.042
*H. uvarum*	CABM8A	MF926284	44.5	–	+	–	–	–	–	0.1	+	Positive with *Botrytis* and *Aspergillus*	0.010
*H. uvarum*	CABCM1A	MF926285	35.8	–	+	–	–	–	–	0.2	–	Positive with *Botrytis*	0.100
*H. uvarum*	CAMM3A	MF926286	34.8	+	+	–	–	–	–	0.1	–	Positive with *Botrytis*	0
*H. uvarum*	CAMM6A	MF926287	40.5	–	–	–	–	–	–	0.3	–	Negative	0.010
*H. uvarum*	SEHI3C	MF927683	25.8	–	–	–	–	–	–	0.1	–	Positeive with *Botrytis*	0.030
*H. uvarum*	SEHI1C	MF926288	21.0	–	+	–	–	–	–	0	+	Positive with *Botrytis*	0.080
*H. uvarum*	SEHM7C	MF926289	26.3	–	–	–	–	–	–	0.1	–	Positive with *Botrytis* and *Aspergillus*	0.150
*H. uvarum*	CAMB9A	MF926290	27.7	–	–	–	–	–	–	0	–	Negative	0.034
*H. clermontiae*	CABMB1A	MF783060	18.7	+	–	–	–	–	–	0	–	Positive with *Botrytis*	0.011
*H. uvarum*	Control	MF801365	28.7	+	–	–	–	–	–	0.3	+	Negative	0.033
M. fructicola^*^	ROMA10	MF783057	28.3	+	–	–	–	–	–	0	–	Positive with *Botrytis*	0.070
*M. guilliermondii*	CABM1A	MF927684	44.5	–	+	–	–	–	–	0.2	+	Negative	0.010
*M. guilliermondii*	SEHIB8	MF783055	37.0	+	+	–	–	–	–	0.2	+	Positive with *Botrytis*	0.027
M. viticola^*^	ROMMA1A	MF783062	46.5	+	–	–	–	–	–	0	–	Positive with *Botrytis*	0.050
*P. kluyveri*	SEHMA6B	MF783059	26.7	–	–	–	–	–	–	0	+	Positive with *Botrytis* and *Aspergillus*	0.014
*P. kluyveri*	CABMC6C	MF926283	29.5	+	–	–	–	–	–	0	–	Positive with *Botrytis*	0.360
*S. cerevisiae*	CABMA3A	MF783053	40.0	–	–	–	–	+	+	0.1	+	Positive with *Botrytis*	0.010
*V. carnescens*	HB02b	MF926293	28.0	–	–	–	–	–	–	0	–	Positive with *Botrytis*	0.110

* *This table shows the most probable yeast strain according to the compared databased belonging to the Metschnikowia clade, but these yeast strains cannot be assigned unequivocally to one of the species in the clade*.

a The percentage is calculated: (M – Mwy)/M*100 where M is the mold growth (cm) without antagonistic yeast on the plate and Mwy is the mold growth in presence of the antagonistic yeast on septet plates (cm). The percentage represents the reduction of mold grown caused by yeast VOCs.

b *Values are expressed in centimeters (diameter of the halo of the positive acetic acid-producing yeast strains on the plate) a strain of Acetobacter was used as positive control*.

c *Positive is when in presence of iron the yeast decreases its antagonistic activity; Negative is when the antagonistic activity of the yeast is the same in presence or in absence of iron*.

d *The values are expressed as the average of the absorbance at 590 nm of three well-subtracted for the control test values*.

**Table 4:** Disease incidence by *A. carbonarius, B. cinerea*, and *P. expansum* after simultaneous inoculation with different concentrations of yeast strains on PDA-agar after 5 days at 25°C under constant light.

**Species**	**Strains**	***A. carbonarius***				***B. cinerea***				***P. expansum***			
		10^6^^*^	**10^5^**	**10^4^**	**10^3^**	**10^6^**	**10^5^**	**10^4^**	**10^3^**	**10^6^**	**10^5^**	**10^4^**	**10^3^**
*A. pullulans*	FZ02a	–	–	–	–	+	–	–	–	+	+	+	+
*C. californica*	CABMC2A	–	–	–	–	+	–	–	–	+	+	+	+
*H. clermontiae*	CABMB1A	+	–	–	–	+	+	+	+	+	+	+	+
*H. uvarum*	SEHMA6A	+	–	–	–	+	+	+	+	+	–	–	–
*H. uvarum*	CABM8A	+	–	–	–	+	+	+	+	+	+	–	–
*H. uvarum*	CABCM1A	+	+	–	–	+	+	+	+	+	+	+	+
*H. uvarum*	CAMM3A	+	+	–	–	+	+	+	+	+	+	+	+
*H. uvarum*	CAMM6A	+	–	–	–	+	+	–	–	+	+	+	+
*H. uvarum*	SEHI1C	+	–	–	–	+	–	–	–	+	+	+	+
*H. uvarum*	SEHM7C	+	–	–	–	+	+	–	–	+	+	+	–
*H. uvarum*	CAMB9A	+	+	+	+	+	+	+	+	+	+	+	+
*H. uvarum*	SEHIC3	–	–	–	–	+	+	+	+	+	+	+	+
*H. uvarum*	Control	–	–	–	–	–	–	–	–	–	–	–	–
*M. guilliermondii*	CABM1A	+	+	–	–	+	+	+	+	+	+	+	–
*M. guilliermondii*	SEHIB8	+	–	–	–	+	+	+	+	+	+	+	+
*P. kluyveri*	SEHMA6B	+	–	–	–	+	+	+	–	+	+	+	+
*P. kluyveri*	CABMC6C	+	+	–	–	+	+	+	+	+	+	+	+
*S. cerevisiae*	CABMA3A	+	+	+	+	+	+	+	+	+	+	+	+
*V. carnescens*	HB02b	–	–	–	–	+	–	–	–	+	+	+	+

*Values are expressed as (+) if yeast strains were able to inhibit the total growth of the mold over a particular concentration and (–) if yeast strains were not able to inhibit mold growth. Values were obtained from three trials*.

* *The values are expressed in CFU/mL*.

#### Killer character assay

From over the 20 yeast strains assayed for the killer character, only *S. cerevisiae* displayed a slightly killer phenotype (Table 3).

#### Enzymatic tests

All yeasts that passed the dual test were evaluated for extracellular enzymatic activities (β-1, 3-glucanase, proteolytic, and pectinolytic activities). Twelve out of the 20 yeast strains were able to hydrolyze at least one of the assayed compound (milk proteins, pectin, glucan, and chitin). Only five yeast strains (4 *M. fructicola*-like yeast strains and 1 *P. kluyveri*) showed all the enzymatic activities (Table 3).

#### Production of volatile organic compounds (VOCs) and hydrogen sulfide release

Percentage data concerning production of VOCs and hydrogen sulfide release among the 20 yeast strains selected showed that 10 yeast strains (3 *H. uvarum*, 4 *M. fructicola*-like yeast strains, 2 *M. guilliermondii*, and 1 *S. cerevisiae*) evidenced the highest values of growth inhibition. These values significantly differed (*p* < 0.05) from the control and the other yeast strains analyzed (Table 3).

#### Biofilm formation

Only yeast strains of *H. uvarum* (1), *P. kluyveri* (1), *V. carnescens*, and *A. pullulans* proved to be able to form biofilm by the adhesion to polystyrene 96-well plate surface (O.D. > 0.1) after 3, 48, and 72 h of incubation (Table 3).

#### Effect of iron concentration on the inhibitory activity of the yeast strains

Antagonistic activity of most of the selected strains were not significantly influenced by tested FeCl_3_ concentrations showing that inhibition activity of these yeasts against *B. cinerea* and *A. carbonarius* were not related with iron competition (Table 3). On the other hand, the activity of the *P. kluyveri* strains resulted iron-sensitive at a concentration of 20 μg/mL of FeCl_3_. The potential yeast strain ROMA10 (*presumably M. fructicola*) always produced red pigments in absence or presence of FeCl_3_ at different concentrations on PDA plates without affecting the pigment coloration or the inhibition of the mold. Regarding the species *A. pullulans*, depending on the concentration of iron, yeast colonies, and haloes pigmentation turned from pale white to maroon, but in absence of FeCl_3_ colonies were not pigmented and the halo was not visible. These findings will be argued in the discussion section.

#### Effect of other metabolites released by yeast strains on mold growth

Yeast primary or secondary metabolism generates numerous compounds as products of the transformation of the carbon, nitrogen, or sulfur sources. Two of the most common substances released are acetic acid and hydrogen sulfide that have antimicrobial effect. Table 3 shows that *M. fructicola*-like strain, *H. uvarum* (2 strains), *M. guilliermondii* (1 strain), *S. cerevisiae*, and *C. californica* species are able to produce these compounds probably affecting the mold development.

### *In vivo* assays for inhibitory activity

#### Efficacy of yeast strains in controlling mold infection on grape berries

The results of the efficacy of the 20 selected strains in reducing molds berry rots are reported in Table 3. *P. kluyveri* (2 strains), *H. uvarum* (2 strains), *H. clermontiae* (1 strain), and *M. guilliermondii* (1 strain) revealed the highest efficacy in reducing mold infection and growth caused by *B. cinerea, A. carbonarius*, and *P. expansum*. On the contrary, a strain of *M. guilliermondii* showed the worst result in controlling molds decay on grape-berries.

#### Comparison of the inhibitory effect with chemical pesticide by *in vivo* test

The three yeast strains which showed a better antagonistic effectiveness against the studied molds taking into account the above described experiments, were subjected to a comparative *in vivo* test with a commercial chemical fungicide used against *B. cinerea* and other molds including *P. expansum* and *A. carbonarius* (Table 5). In this case, the strain *P. kluyveri* SEHMA6B proved to be more effective than the chemical fungicide used under the proposed growth conditions.

**Table 5:** Comparative *in vivo* test of the most suitable yeast strains against molds vs. a commercial chemical fungicide.

**Species**	**Strains**	***A. carbonarius***			***B. cinerea***			***P. expansum***			**Mean**
*H. uvarum*	SEHMA6A	3	3	3	3	3	3	3	3	3	3.00
*H. uvarum*	CABMB9A	2	3	3	3	3	3	3	3	3	2.89
*P. kluyveri*	SEHMA6B	2	2	2	2	1	1	1	3	2	1.78
Commercial fungicide		1	2	2	3	3	3	2	2	3	2.33
Control		4	4	4	4	4	4	3	3	3	3.67

*The disease severity was evaluated by a visual score “1-to-4” (1: no visible symptoms; 2: soft rot; 3: formation of mycelium; 4: sporulation of mold) according to Parafati et al. (2015)*.

## Discussion

The control of fungal diseases and mycotoxins contamination during grape maturation and post-harvesting is currently based on treatments with chemical fungicides. However, the environmental dispersion, the progressive loss of effectiveness, the emergence of resistant strains, and the increasing level of residues in table grape and wine (Marssat et al., 2016), have led the European Union to restrict the use of these compounds, addressing the researchers toward innovative and eco-friendly protocols to face the problem. In agreement with the recommendations pursued by UE Directive 128/2009, this work has been focused on the exploration of the natural antagonistic potential of 241 yeasts isolated from grape samples of *V. vinifera* ssp. sylvestris and *V. vinifera* ssp. vinifera against *B. cinerea, A. carbonarius*, and *P. expansum*. These molds are spoilage agents of the berries, both in vineyard after the veraison and during the over-ripening practices, by rotting the grape bunches that cause the falling of the fruit quality and, in the case of *Aspergillus* and *Penicillium* genera, a threat to food safety due to the release of mycotoxins. According to Wilson and Wisniewski (1989), biocontrol is the application of selected microorganisms with antagonistic activity against other ones and their usage at large-scale to reduce the impact of chemical synthesis pesticides on human health and environment. Many papers report the discovering of novel microbial strains with antifungal properties, proposing them as biocontrol strains against certain molds (Marssat et al., 2016). Although some natural fungicides have been marketed, they can fail in field practices since climatic conditions affect the establishment, survival and activity of the biocontrol agents (Benbow and Sugar, 1999). Yeasts are structurally and functionally heterogeneous because of their differential expression of genes, in a way that epigenetic factors, such as the host environment or abiotic external factors influence the down/up regulation of the gene expression, changing the behavior of yeast populations and their interactions (Spadaro and Droby, 2016). The present investigation shows that yeast strains isolated from various environments have significant differences on the effectiveness against three potentially harmful fungi. To our knowledge, this is the first report in which yeasts isolated from *V. vinifera* ssp. sylvestris and from biodynamic or organic grapevines have been assessed for potential antagonist ability against *A. carbonarius, B. cinerea*, and *P. expansum*.

Our results pointed out that there is a greater number of species found on wildlife vines (23), compared to cultivated ones, with only seven species. This is in line with other studies, which demonstrated that the biodiversity level of yeasts community is influenced by human activities (Cordero-Bueso et al., 2011a,b, 2014, 2017; Martins et al., 2014; Drumonde-Neves et al., 2016). In addition, *S. cerevisae* was also isolated on wildlife grape surfaces. Previous studies on yeast diversity from cultivars or overwintering vines show that *Saccharomyces* genus is either absent on grapes or found in a small number and incidence (Mortimer and Polsinelli, 1999; Torija et al., 2001; Sipiczki, 2016). The results obtained from the preliminary *in vitro* dual assay have clearly disclosed how most isolates collected from wildlife vines (18 strains) are able to inhibit the mold growth vs. the isolates from managed cultivars (only two strains in biodynamic farming). Interestingly, yeast strains, which passed the preliminary tests, have been isolated in two ecosystems where the microbial antagonism against molds could only be produced by the associate microbiota onto grape-berries or natural barriers of the plant that hinder the entry of fungal pathogens. Consequently, *H. uvarum, H. clermontiae, M. guilliermondii*, and *Pichia kluyveri* strains, all of them isolated from *V. vinifera* ssp. sylvestris, could play a pivotal role as biocontrol agents in the natural environment. These data cannot be compared with the current literature since this is the first time that isolates from wildlife vines are studied with this aim. It is possible to hypothesize that the observed differences in microbiota structure between grapes from wildlife vines and cultivated ones can be due to the use of synthetic or natural pesticides in vineyards or the isolation from overwintering vineyards, resulting in a diverse selective pressure on resident microorganisms (Sipiczki et al., 2006 Cordero-Bueso et al., 2011a, 2014; Brysch-Herzberg and Siedel, 2015; Sipiczki, 2016). The higher yeast biodiversity found in samples from native conditions, highlighted in this work, might have been because the natural environment is hostile for the mold development. Moreover, it seems reasonable to think that molds exposed to repetitive doses of synthetic fungicides can acquire, modify, or adjust genetic characters that provide them an increase in the resistance.

The minimum inhibitory concentrations (MICs) assays, defined as the lowest concentrations of yeasts resulting in complete growth inhibition of the molds, have shown that a concentration of 10^5^ cells/mL is enough to reduce the progress of *B. cinerea* and *P. expansum* by all yeast strains. The mold *A. carbonarius* needed a concentration of 10^6^ cells/mL to be inhibited. These concentrations are considerably lower than those found for other antagonistic yeasts (Chanchaichaovivat et al., 2007; Zhang et al., 2007; Nally et al., 2012). However, further experiments are required to evaluate the influence of the growth condition on the MIC values on field.

Since several mechanisms of action are involved in the biocontrol activity of the antagonistic yeasts, we have examined the main modes of actions, such as iron depletion, cell wall degrading enzymes, diffusible, and volatile antimicrobial compounds, and biofilm formation on the 20 selected yeast strains. Within this group *M. guilliermondii, H. clermontiae, P. kluyveri, H. uvarum, A. pullulans*, and the yeast strain ROMA10 (*M. fructicola*-like strain*)* strains proved to release lytic enzymes potentially capable of hydrolyzing the fungal cell wall. Among these species, it is well-known that *A. pullulans* is able to produce β*-*1,3 glucanase, and chitinase active on *Monilinia laxa, B. cinerea*, and *P. expansum*, especially when the mold wall represents the sole carbon source (Zhang et al., 2009).

The yeast metabolism leads to the formation of acetate and ethyl acetate, which are by-products with inhibitory action against molds in storing cereals (Fredlund et al., 2004). Furthermore, some yeasts can emit volatile compounds that inhibit the development of molds, as described by Parafati et al. (2015) where the growth of *B. cinerea* was counteracted by *S. cerevisiae*. In our experimental conditions, the species *H. uvarum, S. cerevisae*, and *M. guilliermondii* were able to release sufficient levels of acetic acid and hydrogen sulfide (evaluated qualitatively) to cause inhibition to mold growth. Likewise, some *M. fructicola-like* strains were capable of preventing the development of molds through the emission of volatile compounds. Regarding this species there are no examples in the literature, despite the report of a commercialized product used as biocontrol agent (Shemer, Bayer CropScience, AG, Germany).

Little is known about the role of biofilms in the biocontrol activity of yeast used to control fungal diseases and the mechanisms involved in their formation. In this work, *H. uvarum, P. kluyveri, V. carnescens*, and *A. pullulans* strains revealed the capability to form biofilm. Previous studies carried on the species *S. cerevisiae* showed that the ability to adhere to a surface was related to the production of extracellular polysaccharides and molecules belonging to glycoproteins family implicated in this action and in the grape wounds protection (Reynolds and Fink, 2001; Parafati et al., 2015). Yeasts cells with the ability to form biofilm are recognized as most effective in limiting pathogen growth being able to colonize more efficiently the inner of grape wounds (Ianiri et al., 2013).

**Figure 2:**
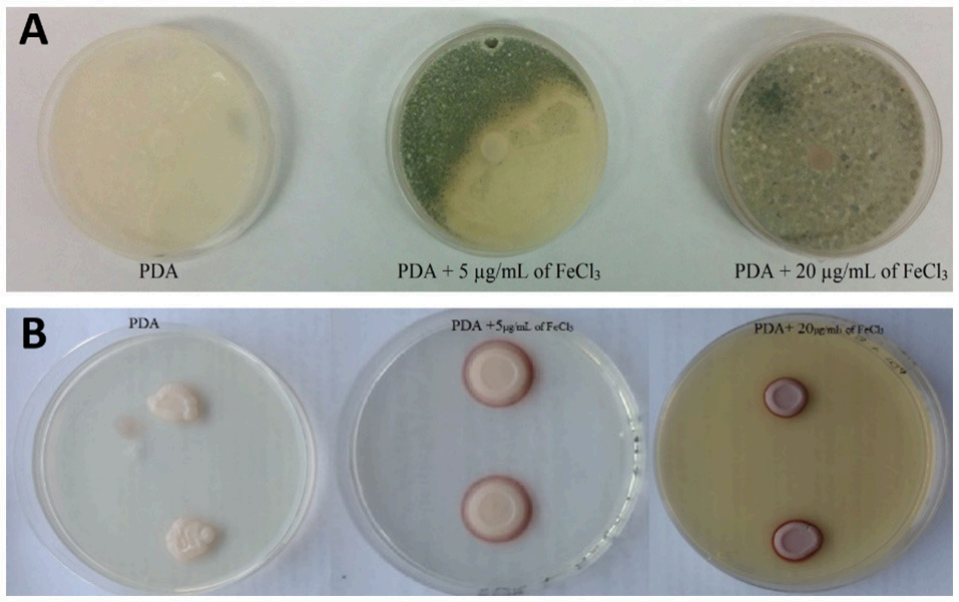
**(A)** Biocontrol activity of *P. kluyveri* SEHMB8A against *P. expansum* in PDA at different concentration of iron. The activity of this yeast strain is iron-sensitive in presence of an excess of iron the strain loses its antagonism. **(B)** Depending on the concentration of iron added to the PDA medium, the species *A. pullulans* turned from pale white to maroon, but in absence of FeCl_3_ the halo was not visible.

**Figure 3:**
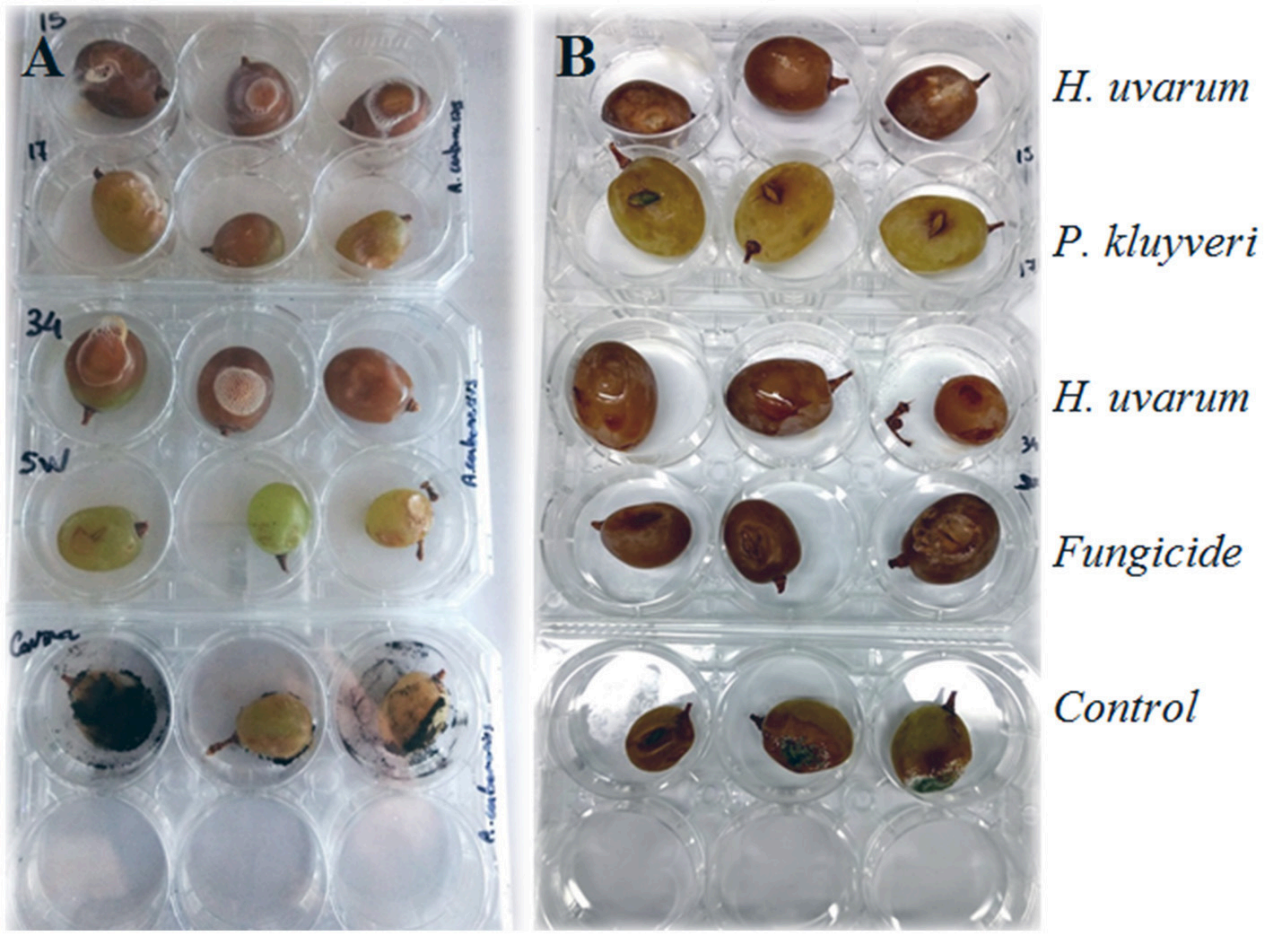
Comparison of the three selected antagonistic yeast strains against *A. carbonarius* **(A)** and *B. cinerea* **(B)** and the commercial fungicide. Line 1: Grapes soaked with *H. uvarum* strain 1, Line 2: Grapes soaked with *P. kluyveri* SEHMB8A, Line 3: Grapes soaked with *H. uvarum* SEHMA61 strain 2, Line 4: Grape soaked with commercial fungicide, Line 5: Grapes without treatment.

Iron is essential for fungal growth and pathogenesis, thus, competition for this metal is functional for counteracting of pathogenic molds. Sipiczki (2006) and Spadaro and Droby (2016) reported this action on strains belonging to the genus *Metschnikowia* that were capable of stopping mold development in crop areas through an iron deficiency mechanism. In the tests we carried out, the presence of iron in growth medium modified the inhibitory properties of the antagonist yeasts (Figure 2A). In particular, for *B. cinerea*, when an excess of iron was present the mold was able to develop contrary to what was happening in growth media without FeCl_3_, where the action of yeast prevented its development. Spadaro and Droby (2016) affirmed that some *M. fructicola* strains were able to produce the red pigment pulcherrimin surrounding its colonies in presence of FeCl_3_ in the growth medium. However, in accordance to Sipiczki (2006), Sipiczki et al. (2013), Brysch-Herzberg and Siedel (2015), Lachance (2016), and Sipiczki (2016) these yeast strains could not be suitable for the delimitation of the species *M. fructicola*. This species is not distinguishable from *M. andauensis* and other species of the *M. pulcherrima* clade because of a possible heterogeneity of the rRNA repeats. Thus, we will consider that these yeast strains are inside of the *M. pulcherrima* clade but not as confirmed *M. fructicola* species. Previous studies investigating the mechanism of antifungal antagonism of pulcherrimin-producing *Metschnikowia* strains claimed that iron immobilization by pulcherrimin (and thus antifungal activity) was suppressed by iron depletion (Sipiczki, 2006). However, in our study, yeast strain ROMA10 (presumably identified as *M. fructicola*) was able to produce pulcherrimin-like substances in presence of FeCl_3_ at the studied concentrations. This result was also previously observed on apple fruits (Saravanakumar et al., 2008). Interestingly, our yeast strain FZ02 identified as *A. pullulans*, did not show halo without the FeCl_3_ addition on the medium, but colonies showed a pink halo at low iron concentration and then they turned to red-maroon at high iron concentrations (Figure 2B). This observation is in accordance with Chi et al. (2013) that reported that in a medium supplemented with iron, the colonies of *A. pullulans* turned to brown. They supposed that the iron was chelated by the secreted siderophores and considerable amount of the intracellular siderophores was responsible for brown colonies. However, further studies are necessary to elucidate both findings described above. The antagonistic potential of the 20 yeast strains selected after *in vitro* tests was further proven on wounded grape berries inoculated with *A. carbonarius, B. cinerea*, and *P. expansum, P. kluyveri, H. uvarum, H. clermontiae*, and *M. guilliermondii* strains exhibited the best efficacy in reducing the development of tested mold diseases. As reported by Parafati et al. (2015), *S. cerevisiae* species reveals to be less efficient than the non-*Saccharomyces* to hamper the fungal growth, probably due to its difficulty to multiply on grape wounds. Nevertheless, these results display that the cumulative effects of different antagonistic activities detected by the *in vitro* tests are not sufficient to explain the outcome of the most performant strains on grape berries (*in vivo* experiments). The efficacy of the yeast strains which showed the greatest *in vivo* action on grape berries, were also compared with a fungicide formulation (37.5% Cyprodinil and 25% Fludioxonil) normally used against *Botrytis* and as secondary rots *Aspergillus* spp. and *Penicillium* spp., according to the supplier's recommendations. We decided to exclude those isolates that show the VOCs production and that release extracellular enzymes, taking into account that the emission of certain compounds, and hydrolytic enzymes by yeasts could alter the balance of the resident microbiota and destabilize the microbial composition of the must. Surprisingly, *P. kluyveri* strain SEHMA6B was more effective than the commercial fungicide, particularly against *Botrytis* (Figure 3). Considering that gray mold decay is the main problem of pre-harvesting, the application of this yeast strain in the field could be even more interesting. Moreover, in a recent study (Sipiczki, 2016) a grape-born *P. kluyveri* strain was tested against *Botrytis* and *S. cerevisiae*. It was active against *Botrytis* but no detectable inhibitory effect on *Saccharomyces*. Other studies have demonstrated that this species is unable to compete with *S. cerevisiae* during fermentation (Cocolin and Ciani, 2014), thus, *P. kluyveri* could be used as biocontrol without alter the fermentation processes. Interestingly, the *P. kluyveri* strain tested by Sipiczki (2016) was isolated from mummified grapes which indicates that it prefers harsh conditions. This fact makes us hypothesize that *P. kluyveri* would be able to cope in the different conditions in field. Nevertheless, further studies are needed to test the antagonistic activity of *P. kluyveri* in field to verify if in the conditions that occur in the vineyard such as temperature swings, high humidity, water, solar radiation, and interaction with the resident microbiota it is able to be effective in counteracting the growth of molds.

Actually, several yeast strains tested in the *in vitro* trials, when air exchange was limited, proved to be effective against molds, while under the *in vivo* outdoor conditions turned out to be ineffective. The main studies on volatile substances are aimed at storing, packaging, and transporting fruit and vegetables (Gomes et al., 2015). From a commercial point of view, it is important to understand the ways in which yeast acts to develop an appropriate formulation and method of application (Spadaro and Droby, 2016). The ability to compete with some nutrient yeast, for example for iron or biofilm formation, is the desired interaction. For these reasons, two isolates of *H. uvarum* and one of *P. kluyveri*, which do not produce hydrolytic enzymes, have been used for the final test with the phytopoietic drug.

Though variable performances in field can be a significant constraint for its practical implementation (Stewart, 2001; Elmer and Reglinski, 2006), the interest in the use of bio-control is renewed because of the recent normative (Directive 2009/128/EC), by matching the specific requirements of International Organization of Vine and Wine for the sustainable production of wine.

In conclusion, this investigation on antagonism patterns in new yeast isolates, over all from *V. vinifera* ssp. sylvestris, can constitute a promising source of knowledge and experience to set strategies in preventing or reducing harvested commodity damages and to test the use of selected yeast strains as a substitutive of the chemical fungicide.

## Author contributions

GC contributed to the design of the work, to the yeast isolation, and identification, to the *in vitro* assays for antagonistic activity, to the analysis and to the interpretation of data for the work, to draft the work and revising it, NM contributed to the *in vitro* assays for antagonistic activity, to *in vivo* assays for inhibitory activity, to draft the work, and revising it, DM to the samples collection for yeast isolation, RF and JC contributed to draft the work and revising it, FV contributed to the yeast identification, IV contributed to the design of the work, to the interpretation of data for the work, to draft the work, and revising it for important intellectual content, and ensured that that questions related to the accuracy or integrity of any part of the work were appropriately investigated and resolved.

### Conflict of interest statement

The authors declare that the research was conducted in the absence of any commercial or financial relationships that could be construed as a potential conflict of interest.
