# Supplementary material for: Wild Grape-Associated Yeasts as Promising Biocontrol Agents against Vitis vinifera Fungal Pathogens
Source: Front Microbiol. 2017 Nov 3;8:2025. doi: 10.3389/fmicb.2017.02025 (PMC5675894; doi:10.3389/fmicb.2017.02025)
Supplement: Supplementary file 1 [file Table1.DOCX]

Supplementary material 2

Table S1. Origin and source of the yeast strains assayed in this study and percentages of the mycelial growth inhibition against *P. expansum*, *B. cinerea* and *A. carbonarius*.

| Species | Strains | Source | Origin | *P. expansum* | | *B. cinerea* | | *A. carbonarius* | | Total |
| --- | --- | --- | --- | --- | --- | --- | --- | --- | --- | --- |
|  |  |  |  | DC: 5.41 | | DC: 8.71 | | DC: 7.51 | |  |
|  |  |  |  | DA | % Inhibition | DA | % Inhibition | DA | % Inhibition |  |
| *A. pullulans* | HULR1C | Wildlife vine | Spain | 0 | 100 | 0 | 100 | 1.5 | 80 | 93.3 |
| *A. pullulans* | FZ02a° | Biodynamic vineyard | Italy | 0 | 100 | 0 | 100 | 0 | 100 | 100 |
| *A. pullulans* | EH02 | Biodynamic vineyard | Italy | 4.4 | 18.5 | 8.7 | 0 | 7.5 | 0 | 6.2 |
| *A. pullulans* | BA02b | Conventional vineyard | Italy | 0 | 100 | 0 | 100 | 2.3 | 69.3 | 89.8 |
| *A. pullulans* | BA02a | Conventional vineyard | Italy | 3.7 | 31.5 | 8.7 | 0 | 7.3 | 2.7 | 11.4 |
| *A. pullulans* | COO1c | Conventional vineyard | Italy | 0 | 100 | 1.1 | 87.4 | 4.4 | 41.3 | 76.2 |
| *A. pullulans* | BV02c | Conventional vineyard | Italy | 5.4 | 0 | 8.7 | 0 | 0 | 100 | 33.3 |
| *C. californica* | CABMC2A | Wildlife vine | Spain | 0 | 100 | 0 | 100 | 0 | 100 | 100 |
| *F. stepposum* | FZ03a | Biodynamic vineyard | Italy | 0 | 100 | 0 | 100 | 0 | 100 | 100 |
| *F. wieringae* | HB09c | Conventional vineyard | Italy | 0 | 100 | 0 | 100 | 0 | 100 | 100 |
| *H. clermontiae* | CABMB1A | Wildlife vine | Spain | 0 | 100 | 0 | 100 | 0 | 100 | 100 |
| *H. pseudoburtonii* | ITORT2A | Wildlife vine | Italy | 1.1 | 79.6 | 0 | 100 | 0 | 100 | 93.2 |
| *H. uvarum* | SEHI1A | Wildlife vine | Spain | 0 | 100 | 0 | 100 | 1.8 | 76 | 92 |
| *H. uvarum* | SEHIC1 | Wildlife vine | Spain | 0 | 100 | 0 | 100 | 0 | 100 | 100 |
| *H. uvarum* | SEHIC3 | Wildlife vine | Spain | 0 | 100 | 0 | 100 | 0 | 100 | 100 |
| *H. uvarum* | SEHMC7 | Wildlife vine | Spain | 0 | 100 | 0 | 100 | 0 | 100 | 100 |
| *H. uvarum* | SEHMA1A | Wildlife vine | Spain | 1.8 | 66.7 | 1 | 88.5 | 1.3 | 82.7 | 79.3 |
| *H. uvarum* | SEHMA6A | Wildlife vine | Spain | 0 | 100 | 0 | 100 | 0 | 100 | 100 |
| *H. uvarum* | CABMB8A | Wildlife vine | Spain | 0 | 100 | 0 | 100 | 0 | 100 | 100 |
| *H. uvarum* | CABMB9A | Wildlife vine | Spain | 0 | 100 | 0 | 100 | 0 | 100 | 100 |
| *H. uvarum* | CABMC5A | Wildlife vine | Spain | 0 | 100 | 0 | 100 | 0 | 100 | 100 |
| *H. uvarum* | CAMM3A | Wildlife vine | Spain | 0 | 100 | 0 | 100 | 0 | 100 | 100 |
| *H. uvarum* | ITARI2A | Wildlife vine | Italy | 0 | 100 | 0 | 100 | 2.9 | 61.3 | 87.1 |
| *H. uvarum* | ROMB3 | Wildlife vine | Romania | 0 | 100 | 7 | 19.5 | 3.2 | 61.4 | 60.3 |
| *H. uvarum* | ROMC4 | Wildlife vine | Romania | 1.8 | 72.3 | 3 | 65.5 | 3.4 | 59 | 65.6 |
| *H. uvarum* | EP02a | Biodynamic vineyard | Italy | 0 | 100 | 0 | 100 | 2.8 | 62.7 | 87.6 |
| *H. uvarum* | FH04 | Biodynamic vineyard | Italy | 0.5 | 90.7 | 0 | 100 | 1 | 86.7 | 92.5 |
| *H. uvarum* | FZ01a | Biodynamic vineyard | Italy | 4.5 | 16.7 | 3.3 | 62.1 | 5.5 | 26.7 | 35.1 |
| *H. uvarum* | EQ02b | Biodynamic vineyard | Italy | 1.8 | 66.7 | 0 | 100 | 1.5 | 80 | 82.2 |
| *Hanseniaspora spp.* | CAMM6A | Wildlife vine | Spain | 0 | 100 | 0 | 100 | 0 | 100 | 100 |
| *Hanseniaspora spp.* | ITSOF2A | Wildlife vine | Italy | 2.5 | 53.7 | 0 | 100 | 2.5 | 66.7 | 73.5 |
| *M. caribbica* | SEHMA2 | Wildlife vine | Spain | 0 | 100 | 0 | 100 | 0 | 100 | 100 |
| *M. fructicola* | ROMA9 | Wildlife vine | Romania | 0 | 100 | 0 | 100 | 2.1 | 74.7 | 91.6 |
| *M. fructicola* | ROMA10 | Wildlife vine | Romania | 0 | 100 | 0 | 100 | 2.7 | 67.5 | 89.2 |
| *M. fructicola* | ROMB6 | Wildlife vine | Romania | 2.3 | 64.6 | 1.2 | 86.2 | 1 | 88 | 79.6 |
| *M. fructicola* | ROMB7 | Wildlife vine | Romania | 1.6 | 75.4 | 0 | 100 | 1.4 | 83.1 | 86.2 |
| *M. fructicola* | ROMB9 | Wildlife vine | Romania | 0 | 100 | 0 | 100 | 0 | 100 | 100 |
| *M. guilliermondii* | BM1 | Wildlife vine | Spain | 0 | 100 | 0 | 100 | 0 | 100 | 100 |
| *M. guilliermondii* | SEHIB8 | Wildlife vine | Spain | 0 | 100 | 0 | 100 | 0 | 100 | 100 |
| *M. pulcherrima* | ITNUR1A | Wildlife vine | Italy | 0 | 100 | 0 | 100 | 1.5 | 80 | 93.3 |
| *M. pulcherrima* | EP02b | Biodynamic vineyard | Italy | 0 | 100 | 0 | 100 | 2.2 | 70.7 | 90.2 |
| *M. pulcherrima* | BX02 | Conventional vineyard | Italy | 2.5 | 53.7 | 0 | 100 | 2.5 | 66.7 | 73.5 |
| *M. viticola* | ITNUR5A | Wildlife vine | Italy | 0.5 | 90.7 | 0 | 100 | 1.5 | 80 | 90.2 |
| *Metschnikowia spp.* | ROMA4 | Wildlife vine | Romania | 0 | 100 | 2.6 | 70.1 | 2.6 | 68.7 | 79.6 |
| *Metschnikowia spp.* | ITFMAGB1C | Wildlife vine | Italy | 0 | 100 | 0 | 100 | 0 | 100 | 100 |
| *P. fermentans* | CABM8C | Wildlife vine | Spain | 0 | 100 | 0 | 100 | 0 | 100 | 100 |
| *P. flavescens* | HB02b | Biodynamic vineyard | Italy | 0 | 100 | 0 | 100 | 0 | 100 | 100 |
| *P. kluyveri* | SEHMA6B | Wildlife vine | Spain | 0 | 100 | 0 | 100 | 0 | 100 | 100 |
| *P. kluyveri* | CABMA4A | Wildlife vine | Spain | 1 | 81.5 | 0 | 100 | 0 | 100 | 93.8 |
| *P. kluyveri* | CABMC6C | Wildlife vine | Spain | 0 | 100 | 0 | 100 | 0 | 100 | 100 |
| *R. babjevae* | SEHM2A | Wildlife vine | Spain | 0 | 100 | 0 | 100 | 1 | 81.5 | 93.8 |
| *R. glutinis* | EP02c | Biodynamic vineyard | Italy | 0 | 100 | 0 | 100 | 2.8 | 62.7 | 87.6 |
| *R. mucilaginosa* | HURM4A | Wildlife vine | Spain | 0 | 100 | 0 | 100 | 0 | 100 | 100 |
| *R. nothofagi* | ITFMAGN3B | Wildlife vine | Italy | 0 | 100 | 0 | 100 | 2.3 | 69.3 | 89.8 |
| *R. paludigena* | SEHMB8B | Wildlife vine | Spain | 0 | 100 | 0 | 100 | 1.5 | 80 | 93.3 |
| *S. cerevisiae* | CABMA3A | Wildlife vine | Spain | 0 | 100 | 0 | 100 | 0 | 100 | 100 |
| *S. stipitis* | SEHM1C | Wildlife vine | Spain | 0 | 100 | 0 | 100 | 0 | 100 | 100 |
| *S. polymorphus* | PIEM5B | Wildlife vine | Italy | 0.5 | 90.7 | 0 | 100 | 0 | 100 | 96.9 |
| *V. carnescens* | HB02c | Biodynamic vineyard | Italy | 0 | 100 | 1.5 | 82.8 | 0 | 100 | 94.3 |
| *V. carnescens* | MI02a | Organic vineyard | Italy | 0 | 100 | 0 | 100 | 5 | 33.3 | 77.8 |
